# RcsB and H-NS Both Contribute to the Repression the Expression of the *csgDEFG* Operon

**DOI:** 10.3390/microorganisms13081829

**Published:** 2025-08-05

**Authors:** Hiroshi Ogasawara, Azusa Tomioka, Yuki Kato

**Affiliations:** 1 Research Center for Advanced Science and Technology, Division of Gene Research, Shinshu University, Ueda 386-8567, Nagano, Japan; azu2f4007sa@gmail.com (A.T.); y.mannpuku@gmail.com (Y.K.); 2 Academic Assembly School of Humanities and Social Sciences Institute of Humanities, Shinshu University, Matsumoto 390-8621, Nagano, Japan; 3 Institute for Fiber Engineering and Science (IFES), Division of Molecules and Polymers, Shinshu University, Tokida 3-15-1, Ueda 386-8567, Nagano, Japan; 4 Renaissance Center for Applied Microbiology, Shinshu University, Nagano-shi 380-8553, Nagano, Japan; 5 Department of Applied Biology, Graduated School of Science and Technology, Shinshu University, Ueda 386-8567, Nagano, Japan

**Keywords:** transcription factor, biofilm formation, curli fimbriae, *Escherichia coli*

## Abstract

Curli fimbriae are a major component of biofilm formation in *Escherichia coli*, and their expression is regulated by numerous transcription factors and small regulatory RNAs (sRNAs). The RcsD-RcsC-RcsB phosphorelay system, which is involved in the envelope stress response, plays a role in this regulation. In this study, we report that DNase-I footprinting analysis revealed that the response regulator RcsB interacts with the −31 to +53 region of the promoter region of *csgD*, which encodes a major regulator of biofilm formation, and thus contributes to its transcriptional repression. Additionally, overexpression of RcsB or RcsB D56A that could not be phosphorylated by the histidine kinases RcsC and D both significantly reduced *csgD* expression and suppressed Curli formation. This indicates that the phosphorylation of RcsB has an insignificant impact on its affinity for its operator sites. Furthermore, we confirm that RcsB binds cooperatively to the *csgD* promoter region in the presence of the nucleoid-associated protein H-NS. Our study also confirms that RcsB positively regulates the expression of an sRNA, RprA, which is known to reduce mRNA *csgD* mRNA translation RprA via its binding to the 5′-untranslated region (UTR) of *csgD*. These findings indicate that, in *E. coli*, the RcsBCD system suppresses *csgD* expression through both direct transcriptional repression by the regulator RcsB and translational repression by the sRNA RprA.

## 1. Introduction

*Escherichia coli* can transition from a single-cell growth form to a biofilm mode in response to environmental changes [1,2]. In the single-cell growth form, *E. coli* develop flagella, which are crucial for cell motility and environmental adaptation. When *E. coli* cells transition from a single-cell planktonic growth mode to a biofilm mode, they inhibit flagella formation and activate gene expression involved in cell–cell adhesion [3,4,5]. The regulation of flagellar formation and cell adhesion in bacteria is influenced by several factors, including transcription factors, small-molecule second messengers, and environmental cues. The biofilm matrix is a complex structure of assembled cells that is responsible for adhesion to abiotic surfaces in the natural environment and on the eukaryotic cellular tissue of host animals and plants [6,7]. In the process of biofilm formation in *E. coli*, Curli fimbriae—as a key factor—play a significant role in the initial step of attachment to solid surfaces and subsequent cell-to-cell adhesion [8,9,10,11]. The *E. coli csgDEFG* operon encodes the transcription factor CsgD [8], the chaperone proteins CsgE [12] and CsgF [13], and CsgG [14], which are transporters of CsgB and CsgA, while the *csgBAC* operon encodes the Curli filament components CsgA, and CsgB, as well as CsgC, which inhibits the polymerization of CsgA and is transcribed in the reverse direction as a divergent operon [15]. CsgD also regulates at least more than 20 target genes, including those involved in motility (*fliE*, *fliFGHIJK*, *fliA*, and *flgM*), the iron-enterobactin transporter (*fepD*), the Curli inhibitor (*csgI,* formally named *yccT*), and several genes with unknown functions [4,16,17]. The transcriptional regulation of the *csgDEFG* operon involves numerous transcription factors that are fine-tuned in response to various environmental conditions (e.g., osmotic pressure, temperature, pH, etc.) [18,19,20,21,22,23]. Since biofilm formation contributes to stress resistance, biofilm development should be optimized in response to different environmental conditions. To do so, multiple transcription factors responding to these diverse environmental conditions contribute to the transcriptional regulation of the *csgDEFG* operon. Previous studies have reported that H-NS [24,25], Crl [8,26], OmpR [18,27,28], CpxR [18,28,29], MlrA [30,31], IHF [19,28], CRP [32,33], RstA [28,34], Cra [35], BasR [36], RcdA [37,38], MqsR [39], and BtsR [40] are involved in *csgD* transcriptional regulation.

PS-TF (Promoter-specific Transcription factor) screening using 198 purified transcription factors out of the total transcription factors in *E. coli* allowed for the identification of 48 TFs (35 known TFs and 13 TFs with unknown functions) that bind strongly to the *csgD* promoter [41]. Positive regulation of *csgD* expression by YhjC (renamed RcdB) and negative regulation by YiaJ (renamed PlaR) were newly identified among the transcription factors of unknown function [41]. In *E. coli* O157: H7 strains, Curli fimbriae formation is regulated by Fis [42], SdiA [43], Hha [44], and QseB [45]. On the other hand, *csgDEFG* expression is regulated by multiple sRNAs [46]. Previous research has reported the involvement of seven sRNAs (RprA [47], GcvB [48], McaS [48], RybB [49], RydC [50], OmrA [51], and OmrB [51]) to be involved, with each sRNA binding to the complementary sequence of the 5′-UTR of *csgD* mRNA and inhibiting CsgD translation. Of these sRNAs, the expression of RprA is regulated by the RcsBCD system (involved in the transcriptional regulation of capsular synthesis genes and flagellar synthesis genes), a known envelope stress response mechanism, in which RprA transcription is activated by phosphorylated RcsB in *E. coli* [52,53]. The histidine kinase RcsC transfers phosphate to RcsD, leading to phosphorylation of RcsB [54]. RcsF is an outer-membrane lipoprotein that plays a crucial role in the Rcs phosphorelay system. In the Rcs system’s inactive state, RcsF resides within the pore of the outer-membrane porin. Conversely, in its active state, RcsF interacts with IgaA, an inhibitor of the Rcs system. This interaction impairs IgaA’s inhibitory function and thus facilitates the phosphorylation of RcsC and the subsequent transfer of phosphate groups to RcsD and RcsB. Phosphorylated RcsB acts independently or in combination with RcsA to positively regulate the transcription of the small RNA (sRNA) RprA, the capsule-encoding genes, and other genes [54].

Unlike RcsB, RcsA lacks an Asp residue in its REC domain, rendering it non-phosphorylatable, despite its structural and domain composition similarities. RcsB, in combination with other FixJ/NarL accessory proteins, also further regulates other functions, independently of RcsB phosphorylation [54]. In *E. coli*, the small RNA RprA binds to the *csgD* mRNA, thereby preventing CsgD translation. Therefore, RcsB indirectly represses *csgD* expression at the posttranscriptional level via the positive regulation it exerts on the expression of the sRNA RprA.

The expression of *csgD* and *csgA* was previously shown to be significantly up-regulated in the *rcsB*-deficient mutant [55,56]. In *Salmonella*, *csgD* translation is also inhibited by RprA, whose expression is stimulated by phosphorylated RcsB, but, in this strain, in contrast to *E. coli*, the expression of CsgD is reduced in a RcsB deletion mutant, and unphosphorylated RcsB induces biofilm formation [57]. These findings suggest that the role of RcsB differs between *E. coli* and *Salmonella*; however, the mechanisms responsible for these differences, including the direct regulation of *csgD* by RcsB, remain to be elucidated. Therefore, in this study, we aimed to investigate the direct and RprA-independent effect of RcsB on *csgD* expression in *E. coli*. We also demonstrated that H-NS plays a role in the regulation of *csgD* expression.

## 2. Materials and Methods

### 2.1. Bacterial Strains and Culture Conditions

The *rprA* or *rprA rcsB* double-mutants were constructed as described in the previous method [58,59], and are listed in Table 1. A DNA fragment in which the chromosomal region adjacent to the *rprA* region was flanked by the CmR cassette from pACYC184 [60] was prepared via PCR using the primers *rprA*-Cm-F and *rprA*-Cm-R. And then the DNA fragment was subsequently introduced by electroporation into *E. coli* MG1655 rsh in which the *recB recC* genes had been replaced by the λ red region. CmR mutant *E. coli* colonies were selected, and the deletion was confirmed by PCR. The P1 phage prepared from the *rprA* deletion mutant with the CmR cassette introduced was transduced into BW25113 and JW2205 strains to construct an *rprA*-deficient strain (AO1 strain) and an *rprA*, *rcsB* double-deficient strain (AO2 strain). *E. coli* BW25113, JW2205 (Δ*rcsB* mutant of BW25113), JW1935 (Δ*rcsA* mutant of BW25113), JW3883 (Δ*cpxR* mutant of BW25113), JW1225 (Δ*hns* mutant of BW25113), AO1 (Δ*rprA* mutant of BW25113), and AO2 (Δ*rprA rcsB* double mutant of BW25113) were cultured in YESCA (yeast extract–Casamino acids) medium with or without L-arabinose at 28 °C with constant shaking at 120 rpm. *E. coli* BL21 (DE3) was used for the expression and purification of RcsB, RcsA, CpxR, H-NS, and RcsB D56N. The DH5α strain was employed for the construction of plasmid DNA.

### 2.2. Plasmid Construction

To construct arabinose-inducible *rcsB*, *rcsF*, or *nlpE* expression plasmids, DNA fragments containing these genes’ coding sequences were prepared by polymerase chain reaction (PCR) using *E. coli* BW25113 genomic DNA as a template, along with a pair of gene-specific primers (see Table 1). Following digestion with EcoRI and XbaI, the PCR-amplified fragment was inserted at the corresponding site of pBAD18 [61] to generate the plasmids pBADrcsB, pBADrcsF, and pBADnlpE. The *rcsB* D56N plasmid, which contains a DNA fragment in which the 56th aspartic acid of *rcsB* is substituted with asparagine, was constructed using pBADrcsB as a template and the mutagenesis primers rcsBD56NF and rcsBD56NR. To construction of an IPTG-inducible *rcsB* D56N expression plasmid, DNA fragments containing the *rcsB* coding sequence were prepared by polymerase chain reaction (PCR) using pBADrcsB D56N as a template, along with the pair of gene-specific primers rcsBF and rcsBR (see Table 1). Following digestion with BamHI and NotI, the PCR-amplified fragment was inserted at the corresponding site of pET21a to generate the plasmid pETrcsBD56N.

**Table 1:** Strains, plasmids, and primers used in this study.

Strain	Genotype	Source or Reference
DH5α BW25113 BL21(DE3) MG1655rsh JW2205 JW1935 JW3883 JW1225 MGDrprA AO1 AO2	F-, 80dlacZΔM15, Δ(lacZYA-argF)U169, deoE, recA1, endA1 hsdR17(rK-,mK+), phoA, supE44, λ-, thi-1, gryA96, relA1 F-, Δ(araD-araB)567, ΔlacZ4787(::rrnB-3), λ-rph-1, Δ(rhaD-rhaB)568, hsdR514 F−, lon-11, Δ(ompT-nfrA)885, Δ(galM-ybhJ)884, λDE3 [lacI, lacUV5-T7 recBCD::red(Km) rpsL hsdR::Ap BW25113 rcsB::kan BW25113 rcsA::kan BW25113 cpxR::kan BW25113 hns::kan MG1655rsh rprA::Cm BW25113 rprA::Cm (BW25113 × P1(MGΔrprA)→Cmr) JW2205 rprA::Cm (JW2205 × P1(MGΔrprA)→Cmr)	TAKARA [62] [63] [64] [59] [59] [59] [59] This study This study This study
**Plasmid**	**Genotype**	**Source or Reference**
pBAD18 pET21a pACYC184 pBADrcsB pBADrcsB D56N pBADrcsF pBADnlpE pKH28-1 pETrcsB D56N	araC, rrnBT, amp lacI, T7promoter/terminator, amp araC, rrnBT, Tet, Cm pBAD18 with fragment containing rcsB ORF pBADrcsB (D56→N56) pBAD18 with fragment containing rcsF ORF pBAD18 with fragment containing nlpE ORF pET21a with fragment containing rcsB ORF pKH28-1 (D56→N56)	[61] Novagen [60] This study This study This study [28] [65] This study
**Primer**	**Sequence (5’-3’)**	**Source or Reference**
rprA-Cm-F rprA-Cm-R F-U500-rprA R-D500-rprA rcsBD56NF rcsBD56NR RCSBF RCSBR rcsF-BAD-EcoRI-F rcsF-BAD-XbaI-R BAD-nlpE-EcoRI-F BAD-nlpE-XbaI-R rcsB-BAD-EcoRI-F rcsB-BAD-XbaI-R csgD-EcoRI-F CD6R-FITC-R BAD-SQ-F2 BAD-SQ-R2 csgD-S csgD-T F-rprA R-rprA csgB-s-N csgB-t-N	CGACGCAAAAAGTCCGTATGCCTACTATTAGCTCACGGAATAAGATCACTACCGGGCG AAAGAGTGAGGGGCGAGGTAGCGAAGCGGAAAAATGTTTAAGGGCACCAATAACTGCC ATTATCTGGCTCTACTGGACTGGCGATACCAC AGCCGCTCCAGATCGTGTGCATATAATTCAGC GATTACCAATCTCTCCATGCCTGGCGATAAGTA GGAGAGATTGGTAATCAACACATGCGCATCCAGT CAAGGTAGCCGGATCCATGAACAATATGAA TATCTGGCCTACAGCGGCCGCGTCTTTATC CGTCTTGGAATTCTTACAAGCTCCTGATT TCTTTATAGGTCTAGAGAATAACGCCTATT CAAGCGTGAATTCGATGCGCGGCAAAGTGC TATATCCTTCTAGACTGTTTTGCGTTTGTT GAATAGAAGAATTCATCAGCGACATTGACA GGTGCAAATTCTAGATAAGACACTAACGCG AGACAGGAATTCTTCTTGCCCGTCGCT GCACTGCTGTGTGTAGTAAT (5′-FITC) ACGGCAGAAAAGTCCACATTGATTATTTGC TTTCACTTCTGAGTTCGGCATGGGGTCAGG TTATCGCCTGAGGTTATCGTTTGC TCTTCAGGCTCTATTATTCTTCTGGATAT TTATAAATCAACATATTGATTTATAAGCATGGAAA AAAAAAAAGCCCATCGTGGGAGATGGGCAA TTTATGATGTTAACAATACTGGGTGCGC TTAACGTTGTGTCACGCGAATAGCCATTT	This study This study This study This study This study This study [65] [65] This study This study [28] [28] This study This study [28] [28] This study This study [28] [28] This study This study [17] [17]
cpxP-F cpxP-R gadA-F gadA-R	TCAACGCTGGCAGTCAGTTCATTAA GGAACGTGAGTTGCTACTACTCAATA ATGGACCAGAAGCTGTTAACGGA TGCCAGCAGATTTGTACCGGA	[28] [28] [66] [66]

### 2.3. Expression and Purification of Transcription Factors

The expression and purification of transcription factors (TFs) of *E. coli* K-12 were conducted using the standard system [65]. Histidine-tagged TFs were expressed in *E. coli* BL21(DE3) transformed with RcsB, RcsA, and RcsB D56N expression plasmid [65]. The purity of TFs used in this study was checked using SDS-PAGE.

### 2.4. Gel-Shift Assay

Probes containing the FITC-labeled DNA fragments, including the *csgD* promoter, were generated using PCR amplification using a pair of primers (csgD-EcoRI-F; 5′-AGACAGGAATTCTTCTTGCCCGTCGCT-3′ and CD6R-FITC-R; 5′-GCACTGCT GTGTGTAGTAAT-3′) and the plasmid pRScsgD containing a DNA fragment from −335 to +67 relative to the *csgD* transcriptional start site as the template, along with Blend Taq DNA polymerase (TOYOBO, Osaka, Japan). PCR products with FITC at the 5′-termini were purified using PAGE. Mixtures of FITC-labeled probes and purified RcsA, H-NS, RcsB, and RcsB D56N mutant were subjected to a gel-shift assay under standard conditions [28,31].

### 2.5. DNase I Footprinting Assay

The DNase I footprinting assay was conducted as described in a previous study [28]. FITC-labeled DNA probes (0.5 pmol each) were incubated at 37 °C for 30 min, with varying concentrations of RcsB or RcsB D56N in binding buffer [25 μL of 10 mM Tris/HCl (pH 7.8), 150 mM NaCl, 3 mM magnesium acetate, 5 mM CaCl_2_, and 25 μg BSA mL^−1^]. Following a 5 min incubation at 25 °C, DNA digestion was initiated by the addition of 6.25 ng of DNase I (Takara Bio, Kusatsu, Japan). After 30 s of digestion at 25 °C, the reaction was terminated by the addition of 25 μL of phenol. The digested DNA fragments were precipitated with ethanol, dissolved in formamide-dye solution, and analyzed using electrophoresis on a 6% polyacrylamide gel containing 7 M urea using the DSQ-2000L DNA sequencer (SHIMADZU, Kyoto, Japan).

### 2.6. Northern Blotting Assay

Northern blotting was conducted as previously described [28]. For the preparation of total RNA, overnight cultured cells were grown in 30 mL of YESCA medium supplemented with L-arabinose (final concentration 0.2%) at 28 °C and 140 rpm for 12 h. DIG-labeled DNA fragments were amplified by PCR using BW25113 genomic DNA (50 ng) as a template, DIG-11-dUTP (Roche, Basel, Switzerland) and dNTPs as substrates, each gene-specific forward and reverse primers (Table 1), and Blend Taq DNA polymerase (TOYOBO, Osaka, Japan). Each of 6 μg of total RNA was denatured by incubation at 65 °C for 10 min in formaldehyde-MOPS gel loading buffer, separated by electrophoresis on a 2% agarose gel containing formaldehyde, and transferred to a nylon membrane (Roche). Hybridization with the DIG-labeled DNA fragments was performed overnight at 50 °C using the DIG easy Hyb system (Roche). For the detection of the DIG-labeled DNA fragments, the membrane was treated with anti-DIG-AP Fab fragment and CDP-Star (Roche), and the image was scanned with a Typhoon Trio (GE Healthcare, Chicago, IL, USA).

### 2.7. Congo Red Plate Assay to Detect Curli Formation

A Congo red plate assay was performed as described in a previous method [67]. *E. coli* strains were grown at 28 °C for 48 h on YESCA plates containing 50 μg/mL Congo red and 10 μg/mL Coomassie blue.

## 3. Results

### 3.1. Regulatory Impacts of Various Regulatory Genes on csgD, rprA and gadA Expression

#### 3.1.1. Regulatory Role of RcsB, RprA, and RcsF on csgD and rprA Expression

**Figure 1:**
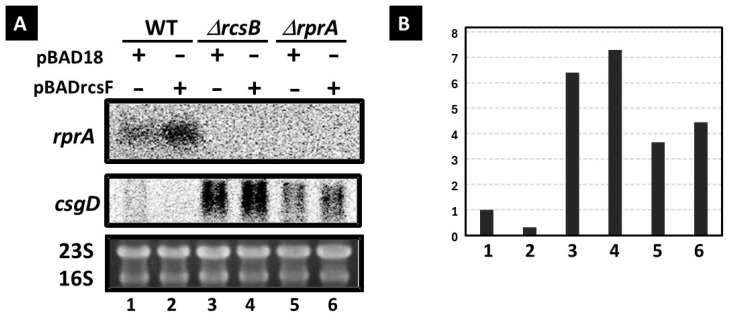
Effects of RcsF overexpression on *csgD* expression in various deletion mutants. (**A**) *E. coli* wild-type (BW25113, lanes 1 and 2), *rcsB* mutant (JW2205, lanes 3 and 4), and *rprA* mutant (AO1, lanes 5 and 6), transformed with the control plasmid (pBAD18, lanes 1, 3, and 5) or the RcsF expression plasmid (pBADrcsF, lanes 2, 4, and 6), were cultivated in YESCA medium at 28 °C for 12 h in the presence of 0.2% L-arabinose. Total RNA isolated from each culture was analyzed using Northern blotting. The *rprA* (positive control as the target gene of RcsB) and *csgD* mRNA were detected using DIG-labeled *rprA* and csgD 3′ probes. The 23S and 16S rRNA (lower panel) were visualized using ethidium bromide staining. Figure (**B**) shows the quantification of band intensity, normalized to the csgD mRNA level in the wild-type strain containing the control plasmid [lane 1 in (**B**)].

To investigate the potential influence of RcsB on the in vivo expression of *csgD*, a Northern blotting assay was conducted using the *E. coli* K-12 wild-type strain (BW25113) and its *rcsB* or *rprA* deletion mutants. To confirm the direct regulatory role of RcsB on *csgD* transcription, RcsF was supplied in trans by introducing an arabinose-inducible plasmid for RcsF expression in these strains. The expression of *csgD* in the wild-type strain decreased following RcsF expression (Figure 1, lane 2), whereas *csgD* expression in the *rcsB* mutant was markedly up-regulated regardless of RcsF induction (Figure 1, lanes 3 and 4). Previous studies have reported that the translation of CsgD in *E. coli* is repressed by the small RNA *rprA* at the post-transcriptional level in *E. coli*, with *rprA* gene expression being activated by phosphorylated RcsB. Indeed, *csgD* expression was induced in the *rprA* mutant; however, the level of *csgD* induction in the *rcsB* strain was higher than that observed in the *rprA* deletion mutant (Figure 1, lanes 3, 4, 5, and 6). These findings suggest a dual function for RcsB: repressing *csgD* transcription by directly binding to the *csgD* promoter, and repressing CsgD translation by activating *rprA* expression through phosphorylated RcsB.

#### 3.1.2. Influence of RcsB Phosphorylation on the Expression of the csgDEFG and csgBAC Operons and on Curli Formation

**Figure 2:**
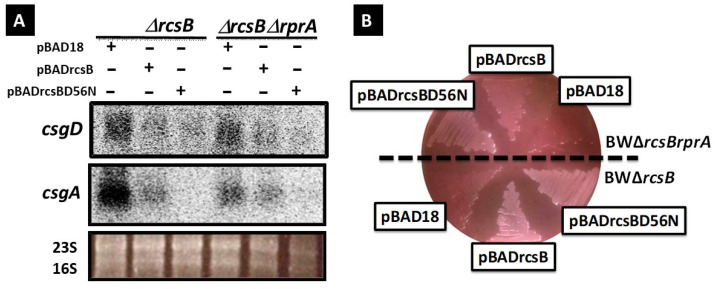
Effects of RcsB phosphorylation on *csgD* and *csgB* expression. (**A**) *E. coli rcsB* mutant (JW2205, lanes 3, 4, and 5) and *rcsB rprA* double-mutant (lanes 6, 7, and 8) transformed with the control plasmid (pBAD18, lanes 1 and 4). RcsB expression plasmid (pBADrcsB, lanes 2 and 5), and RcsB D56N mutant expression plasmid (pBADrcsB-D56N, lanes 3 and 6) were grown in YESCA medium at 28 °C for 16 h in the presence of 0.2% L-arabinose. Total RNA isolated from each culture was detected using Northern blotting. The *csgDEFG* and *csgBA* mRNA were detected using DIG-labeled *csgD* (upper panel) and *csgA* (middle panel) 3′ probes. The 23S and 16S rRNA (lower panel) were detected using ethidium bromide staining. (**B**) Inhibition of curli fimbriae formation through RcsB or RcsB D56N mutant overproduction. *E. coli rcsB* mutant (lower panel), and *rcsB rprA* double mutant (upper panel) transformed with the control plasmid (pBAD18). RcsB expression plasmid (pBADrcsB) and RcsB D56N mutant expression plasmid (pBADrcsB-D56N) were incubated on a Congo red YESCA plate at 28 °C for 48 h in the presence of 0.2% L-arabinose.

The potential influence of unphosphorylated RcsB on the expression of curli genes (*csgDEFG* and *csgBAC*) and curli synthesis was investigated. Northern blotting and Congo red staining assays (quantification of curli synthesis) were performed on rcsB-deletion or *rcsB rprA* double-deletion mutants transformed with either a wild-type RcsB or RcsB D56A mutant (that cannot be phosphorylated) expressed from an inducible promoter present in the over-expression plasmid. Northern blotting analysis revealed a significant decrease in *csgD* and *csgB* expression in the *rcsB*-deletion and *rcsB rprA* double-deletion mutant strains complemented by wt RcsB or mutant RcsB D56N (Figure 2A). This decrease seemed slightly more intense in the two strains containing the overexpression plasmid carrying RcsB D56A than in those containing the plasmid carrying wt RcsB. In the Congo red staining assay, *E. coli* cells were grown for two days on YESCA–Congo red plates, and the color of the colonies was assessed. Control vector plasmid-transformed strains formed red colonies, whereas strains overexpressing RcsB or RcsB D56N from the plasmid did not stain with Congo Red (Figure 2B), consistent with Northern blot results. These findings clearly demonstrate that RcsB regulates negatively the expression of the *csgDEFG* and *csgBAC* operons, and thus curli fimbriae formation, in both its phosphorylated and unphosphorylated states.

#### 3.1.3. Impact of Mutants of the Transcription Factors RcsB, RcsA, CpxR, and H-NS and of RcsF Overexpression on csgD and gadA Expression

**Figure 3:**
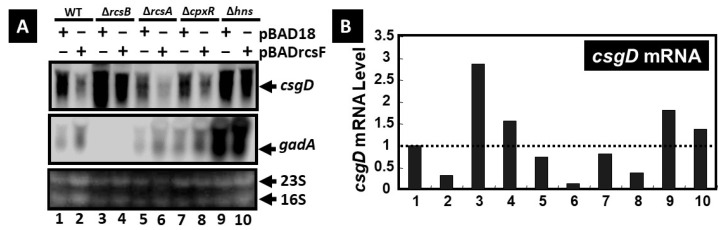
Northern blot analysis was conducted to assess the expression levels of *csgD* mRNA. Panel (**A**) presents data from various *E. coli* strains. The analysis included the *E. coli* wild-type strain (BW25113, lanes 1 and 2), *rcsB* deletion strain (JW2205, lanes 3 and 4), *rcsA* deletion strain (JW1935, lanes 5 and 6), *cpxR* deletion strain (JW3883, lanes 7 and 8), and *hns* deletion strain (JW1225, lanes 9 and 10). Each strain contained either a control plasmid vector (pBAD18, lanes 1, 3, 5, 7, and 9) or an RcsF expression plasmid (pBADrcsF, lanes 2, 4, 6, 8, and 10). Cultures were grown in YESCA medium at 28 °C for 12 h in the presence of 0.2% arabinose. Total RNA was extracted from each culture, fractionated by agarose gel electrophoresis, and subjected to Northern blotting. Detection of *csgD* and *gadA* mRNA was performed using DIG-labeled *csgD* (upper panel) or *gadA* (middle panel) probes, while 23S and 16S rRNA were visualized by ethidium bromide staining (lower panel). Panel (**B**) shows the quantification of band intensity, normalized to the *csgD* mRNA level in the wild-type strain containing the control plasmid [lane 1 in (B)].

The impact of RcsF over-expression on the transcription of *csgD* and *gad* A was also investigated using Northern blot analysis in mutants of the transcription factors RcsB, RcsA, CpxR, and H-NS. In the wt strain, in the *rcsA* deletion strain, and in the *cpxR* deletion strain (to a lesser extent), the over-expression of RcsF led to a reduction in *csgD* expression and to an enhancement of *gadA* expression. This suggested that RcsF had a negative impact on *csgD* expression and a positive one on *gadA* expression. In contrast, the expression of *csgD* was greatly enhanced in the *rcsB* deletion strain, containing or not containing RcsF, in comparison with the wt strain, whereas the expression of *gadA* was undetectable in this strain. This indicated that RcsB, as RcsF, has a negative impact on *csgB* expression and positive impact on gadA expression. Interestingly the expression of both *csgD* and *gadA* was strongly enhanced in the hns mutant in comparison to the wt strain. This indicated that H-NS represses the transcription of these genes (Figure 3). In addition, in the *hns*-deletion strain, elevated levels of RcsF expression did not inhibit *csgD* (Figure 3).

### 3.2. Both Phosphorylated and Unphosphorylated RcsB Interacts with the Promoter Region of csgD In Vitro

**Figure 4:**
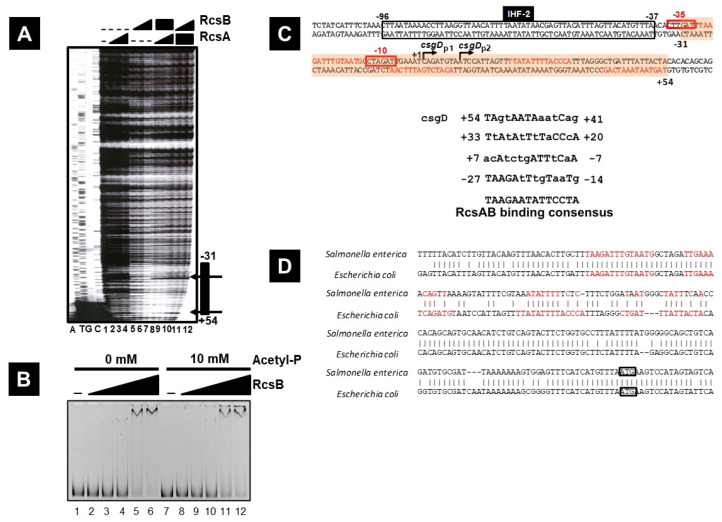
DNase I footprinting and gel-shift assay for transcription factor binding sites on the *csgD* promoter. (**A**) The FITC-labeled *csgD* promoter fragment (1.0 pmol) was incubated in the absence (lane 1) or presence of increasing concentrations of purified RcsB or RcsA (lanes 2–10) and then subjected to DNase I footprinting assays. The concentrations of RcsA used were 10 and 20 pmol for lanes 2 and 3, and 40 pmol for lanes 4 and 8–10, while the concentrations of RcsB used were 20, 40, and 80 pmol for lanes 5–7, and lanes 8–10. Each A, T, G, and C represents the sequence ladders. The numbers indicate the distances from the *csgD* transcription initiation site P1. (**B**) The FITC-labeled *csgD* promoter fragment (0.5 pmol) was incubated in the absence (lane 1) or presence of increasing concentrations of purified RcsB. The concentrations of RcsB used were 0, 2.5, 5.0, 10, 20, and 40 pmol for lanes 1–6, and lanes 7–12. In this experiment, acetyl phosphate was employed at the same concentration utilized in the previous study [69]. Acetyl phosphate (10 mM) was used for the phosphorylation of RcsB (for lanes 7–12). (**C**) Locations of the RcsB or RcsA–RcsB complex binding sites on the *csgD* promoter. The RcsB binding site was identified using DNase I footprinting, revealing four RcsB sites (Rcs1, 2, 3, and 4). The RcsB binding consensus sequence on the *csgD* promoter is shown under the nucleotide sequence of the *csgD* promoter region, compared with the RcsAB on the *flhDC* promoter region. (**D**) The *E. coli csgD* promoter sequence upstream of the *csgD* start codon and the locations of RcsB binding sites (indicated in red) are shown on the lower sequence, while the sequence for the *Salmonella csgD* promoter sequence and the location of the RcsB binding site (indicated in red) are shown in the upper sequence.

The binding activity of RcsB to the *csgD* promoter was examined in vitro using RcsB fused with a 6× His tag at the C-terminus. The gel-shift assay demonstrated the binding of His-tagged RcsB to the *csgD* probe. Notably, the binding of RcsB to the *csgD* promoter fragment was unaffected by the presence or absence of acetylphosphate (Figure 4B). Purified RcsB D56N has also been shown to bind to the *csgD* promoter fragment (Supplemental Figure S1A).

RcsB is been proposed to bind to a region containing an RcsA/B complex binding consensus sequence, consisting of a 14-nucleotide motif (TAAGAATATTCCTA) at 13 bp intervals in the promoter region of *flhD*, one of the previously identified targets of RcsB [68]. A search for this sequence in the *csgD* promoter region, newly identified by DNase I footprinting, revealed four consensus motifs as potential recognition targets of RcsB using the MEME (Multiple EM for Motif Elicitation) program (http://meme-suite.org/tools/meme accessed on 11 May 2019) (Figure 4A,C). These RcsA/B-binding consensus sequences were located around the *csgD* transcription start site (from −31 to +54) (Figure 4A,C).

### 3.3. Both RcsB and H-NS Negatively Regulate csgD Expression

**Figure 5:**
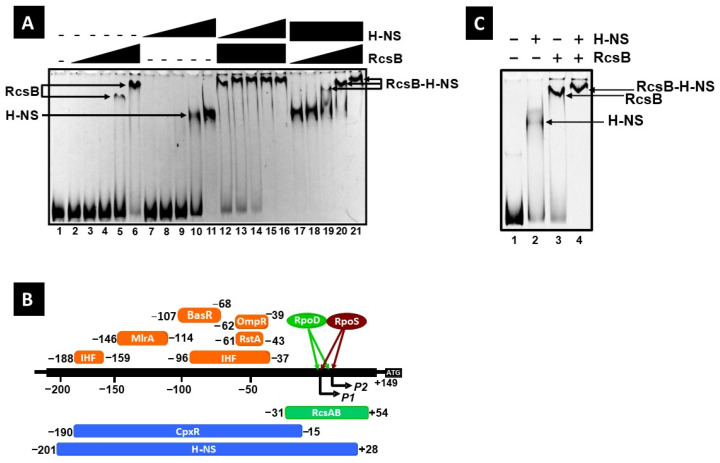
Cooperative binding of RcsB and H-NS to the *csgD* promoter. (**A**) A gel-shift assay was performed using RcsB and H-NS with the FITC-labeled *csgD* promoter fragment (0.5 pmol). The concentrations of RcsB employed were 2.5, 5.0, 10, and 20 pmol for lanes 2–5 and 17–20 and 40 pmol for lanes 6, 12–16, and 21. The concentrations of H-NS used were 2.5, 5.0, 10, and 20 pmol for lanes 7–10 and 12–15 and 40 pmol for lanes 11, 16, and 17–21. (**B**) Locations of the transcription factor binding sites on the *csgD* promoter. The binding sites for OmpR, RstA, CpxR, IHF, MlrA, BasR, and H-NS were identified using DNase I footprinting assays in a previous study, while that of RcsB was newly identified in this study. These binding sites are illustrated along the *csgD* promoter sequence. The numbers at the ends of each line indicate the distance from the P1 transcription start site [8]. (**C**) A gel-shift assay was performed using RcsB and H-NS with the FITC-labeled *csgD* promoter fragment (0.5 pmol). The concentrations of RcsB employed were 20 pmol for lanes 3 and 4. The concentrations of H-NS used were 20 pmol for lanes 2 and 4.

The mRNA levels of *csgD* significantly increased in a mutant lacking RcsB (see Figure 1), consistent with its role as a negative regulator [70]. Since both RcsB and H-NS bind to extensive and overlapping regions, from −31 to +54 for RcsB and −201 to +28 for H-NS (Figure 5B), a DNA fragment encompassing the −335–+67 region from the *csgD* transcriptional start site was used for EMSA. Both RcsB and H-NS independently formed complexes with the FITC-labeled *csgD* probe (Figure 5A, lanes 1–11). In the simultaneous presence of both RcsB and H-NS, the DNA complexes exhibited super shifting (Figure 5A, lanes 12–21), indicating that both factors can bind to the DNA probe, despite the substantial overlap of their binding sites. This observation suggests a cooperative repression of the *csgD* promoter by the two negative factors, RcsB and H-NS.

The level of CsgD is thus regulated by cooperative and/or competitive interactions between various transcription factors, including RcsB and H-NS, each responding to different environmental conditions.

## 4. Discussion

In this study, we demonstrate that, in *E. coli*, the repression of *csgD* expression by the Rcs surface stress response pathway involves the transcriptional repression of *csgD* expression through direct binding of RscB to the *csgD* promoter region, as well as the translational repression of *csgD* mRNA by sRNA RprA, whose expression is under the positive control of the phosphorylated regulator RcsB. The transcriptional regulation of the *csgDEFG* operon, which is needed for Curli formation in *Escherichia coli* [3,22], involves multiple transcription factors. Previous research demonstrated the involvement of several two-component systems (TCSs) in this regulation, such as EnvZ/OmpR [18,19], RstB/RstA [28,34], and BasS/BasR [36], which positively regulate the expression of the *csgDEFG* operon, whereas CpxA/CpxR [70], RcsC/RcsD/RcsB [47,55], and BtsS/BtsR [40] negatively regulate *csgDEFG* expression. In *E. coli*, Rcs and Cpx are the major TCSs involved in the protection of cells from envelope stress and which facilitate their adaptation to this stress. These systems include the outer-membrane lipoproteins RcsF and NlpE, which act as sensors [71]. In this study, we demonstrate that RcsB binds to the −30–+54 region of the *csgDEFG* promoter (Figure 4) and thus overlaps, from −31 to +28, with the H-NS binding site located between −201 and +28 of the csgD transcriptional start site. This suggested a possible cooperative binding for the repression of *csgDEFG* expression.

Similarly, Ogasawara et al., 2010 showed previously that H-NS and CpxR cooperatively bind the *csgD* promoter region to repress *csgDEFG* expression. In the wild-type *E. coli* strain, where the outer membrane lipoprotein NlpE is over-expressed and the Cpx system is activated, *csgDEFG* is repressed in a CpxR- and H-NS-dependent manner [28]. However, in the *rcsB*-mutant strain, *csgDEFG* is expressed at the same level as in the wild-type strain, confirming that the transcriptional regulation of *csgD* by the Cpx and Rcs systems operates independently of each other (Supplemental Figure S2).

The expression of the *flhDC* operon, which encodes the flagellar master regulator, is cooperatively repressed by RcsB and RcsA. RcsB was shown to bind to both the template (from +5 to +25) and non-template (from +2 to +22) strands downstream of the *flhDC* transcription start site [68]. The region from +5 to +18 contains the RcsA/RcsB binding consensus sequence (Figure 4C). RcsB binds to these regions at lower concentrations when RcsA is present compared to when it is absent, indicating that RcsA facilitates RcsB binding [68]. In this study, the binding site of RcsB was identified between positions −31 and +54 from the transcriptional start site of the *csgD* promoter P1. In the presence of RcsA, a significant reduction in DNase I footprinting ladders, including between positions +20 and +54 from the *csgD* transcription start site, was observed, indicating strong binding of RcsB to this region (Figure 3A). The most downstream RcsB common sequence (from +41 to +54) did not overlap with the H-NS binding site of the *csgD* promoter and exhibited higher homology with the +5–+18 sequences on the *flhDC* promoter than other RcsB common sequences (from +20 to +33, −7 to +7, and −27 to −14), suggesting the RcsA/RcsB-dependent binding of this region (from +41 to +54, downstream of the *csgD* promoter), as in the RcsA/RcsB-dependent *flhDC* promoter region. However, the deletion of *rcsA* was shown to have no impact on *flhDC* expression (Figure 5).

RcsB regulates the transcription of target genes, either as a homodimer or as heterodimer in conjunction with coregulators such as RcsA, BglJ, GadE, MatA, and DctR [72,73,74,75,76].

The formation of RcsB heterodimers suggests that both the target gene and the RcsB auxiliary regulators may themselves be subject to repression by H-NS. Consequently, induction may necessitate the alleviation of H-NS repression [74,77,78,79,80].

H-NS repression is frequently observed in recently acquired genes; it is probable that many of these auxiliary proteins have also been recently acquired, as they are only found in a subset of the numerous species that express RcsB and the Rcs phosphorelay [54]. A previous study indicated that H-NS binds at −201–+28 in the *csgDEFG* promoter [28]. Since *rcsA* transcription was shown to be repressed by H-NS [81], the influence of RcsA on the regulation of *csgD* expression is likely to be limited. Interestingly, the over-expression of RcsF in an *hns* deletion strain showed almost no reduction in the level of *csgD* transcripts (Figure 3). This suggested that coordinated binding by RcsB and H-NS to the *csgD* promoter region may be responsible for the repression of *csgD* expression (Figure 5).

**Figure 6:**
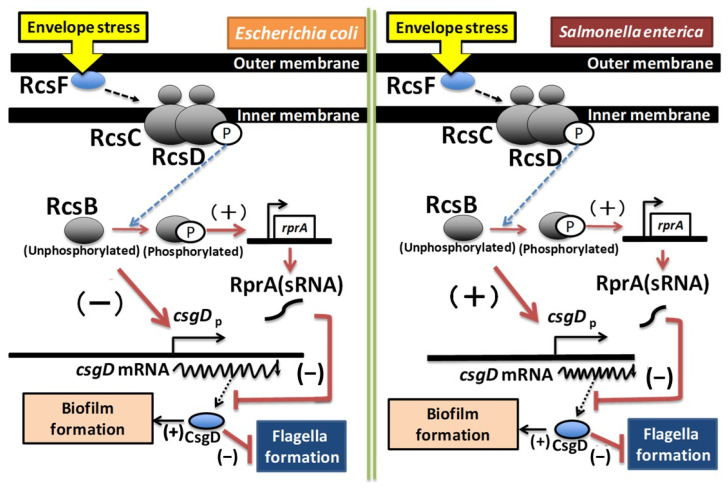
The models of the regulation of *csgD* expression by RcsB in *E. coli* and *Salmonella*. The left panel shows the regulation of *csgD* expression in *E. coli* and the right panel shows the regulation of *csgD* expression in *Salmonella*.

Furthermore, the regulation of *csgD* expression by RcsB exhibits significant differences between *E. coli* and *Salmonella*. In *Salmonella*, the deletion of RcsB results in a marked reduction in *csgD* expression [57], whereas in *E. coli* it leads to a significant increase [55]. An analysis of the *csgD* promoter regions in *E. coli* and *Salmonella* reveals a high sequence homology upstream of the *csgD* ORF start codon. However, the sequence from the transcription start site *csgD* P1 to +54 in *E. coli* shows low homology with the corresponding *Salmonella csgD* promoter sequence (Figure 4D). These sequences contain RcsB binding consensus sequences and sRNA-binding regions, which may account for the observed differences in RcsB’s regulatory effects on *csgD*. Additionally, the influence of H-NS on *csgD* expression also varies between *E. coli* and *Salmonella* [19,23,25]. This variation may be related to differences in the promoter region sequences, necessitating further investigation (Figure 6). In *E. coli*, RcsB represses *csgD* expression, irrespective of its phosphorylation status, leading to the induction of Curli formation and capsule synthesis [55]. Conversely, in Salmonella, Curli formation is induced in a manner dependent upon non-phosphorylated RcsB, and *rcsB* deletion results in the suppression of both Curli formation and capsule synthesis [57]. It is plausible that alterations in the RcsB binding site within the *csgD* promoter sequence may modify the regulation of Curli formation, thereby affecting biofilm formation differently in *E. coli* and *Salmonella*. However, the binding of RcsB to the *csgD* promoter has not been confirmed yet in *Salmonella* [57], and the regulatory mechanism remains unknown, necessitating further investigation.

## 5. Conclusions

In our study, we demonstrate, for the first time, that RcsB and H-NS binds cooperatively at specific sequences present in the *csgD* promoter and represses *csgD* transcription. In summary, we propose that, in *E. coli*, the Rcs system controls curli and biofilm formation via the RscB-mediated direct negative regulation of the expression of the *csgDEFG* and *csgBAC* operons, as well as by the RscB-mediated positive regulation of the expression of sRNA RprA that impairs *csgD* translation via its pairing with *csgD* mRNA.

## Data Availability

The original contributions presented in this study are included in the article/Supplementary Material. Further inquiries can be directed to the corresponding author.

## Supplementary Materials

The following supporting information can be downloaded at: https://www.mdpi.com/article/10.3390/microorganisms13081829/s1, Figure S1: Gel-shift assay and DNase I footprinting analysis of the interaction between purified RcsB D56N and the csgD promoter; Figure S2: The effect of CpxP overexpression was assessed using Northern blot analysis to determine the level of csgD mRNA expression.
