# Supplementary material for: RcsB and H-NS Both Contribute to the Repression the Expression of the csgDEFG Operon
Source: Microorganisms. 2025 Aug 5;13(8):1829. doi: 10.3390/microorganisms13081829 (PMC12388358; doi:10.3390/microorganisms13081829)
Supplement: Supplementary file 1 [file microorganisms-13-01829-s001.zip › microorganisms-3776218-supplementary.pdf]

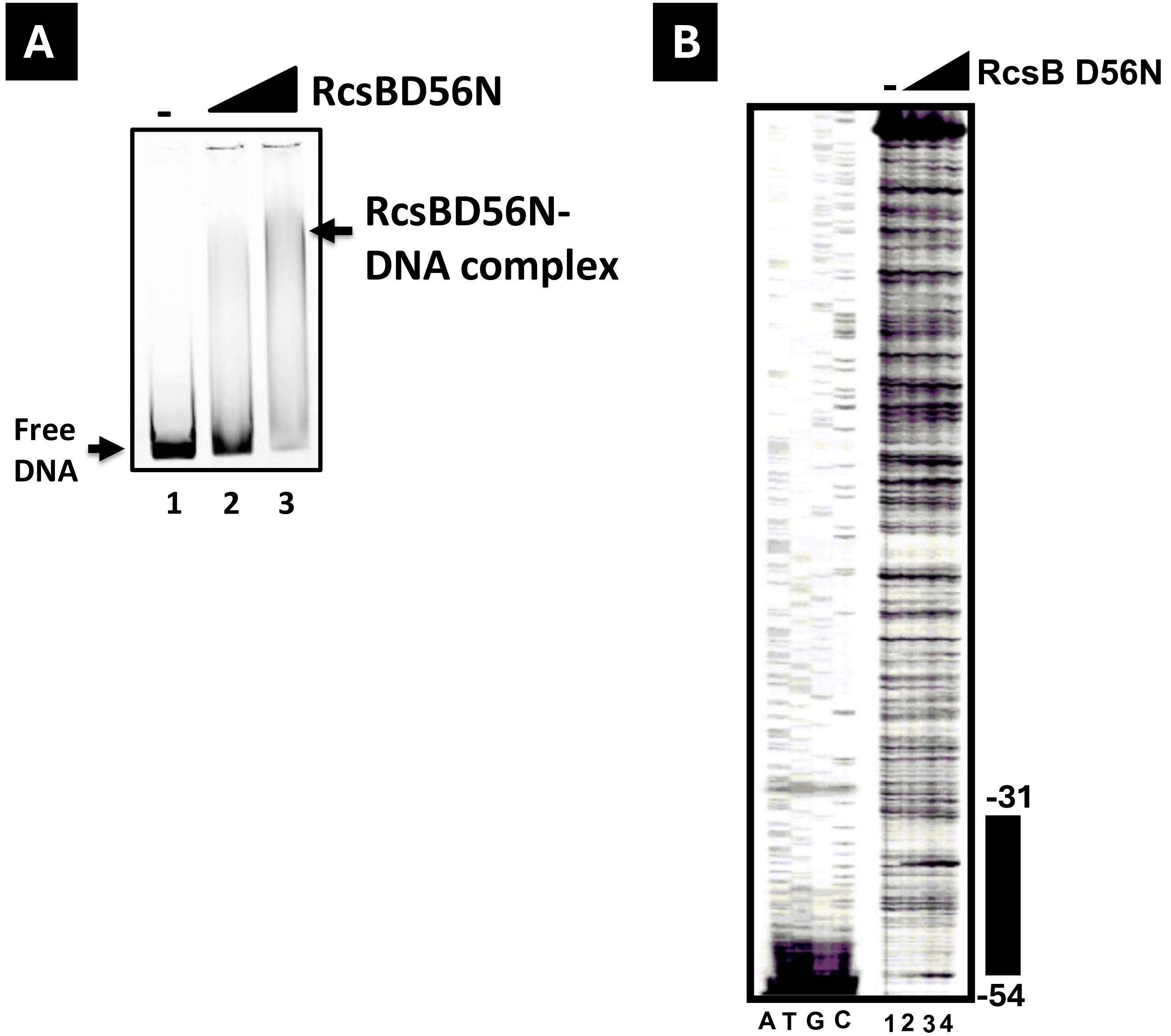

Supplemental Figure S1. Electrophoretic mobility shift assay (EMSA) and DNase I footprinting analysis of the interaction between purified RcsB D56N and the *csgD* promoter. (A) The fluorescein isothiocyanate (FITC)-labeled *csgD* promoter fragment (0.5 pmol) was incubated either in the absence (lane 1) or in the presence of increasing concentrations of purified RcsB D56N. The concentrations of RcsB D56N employed were 0, 10, and 20 pmol for lanes 1 through 3, respectively. (B) The FITC-labeled *csgD* promoter fragment (1.0 pmol) was incubated either in the absence (lane 1) or in the presence of increasing concentrations of purified RcsB D56N (lanes 2-4), followed by DNase I footprinting assays. The concentrations of RcsB D56N used were 10, 20, and 40 pmol for lanes 2 through 4, respectively. Each A, T, G, and C denotes the sequence ladders. The numbers indicate the distances from the *csgD* transcription initiation site P1.

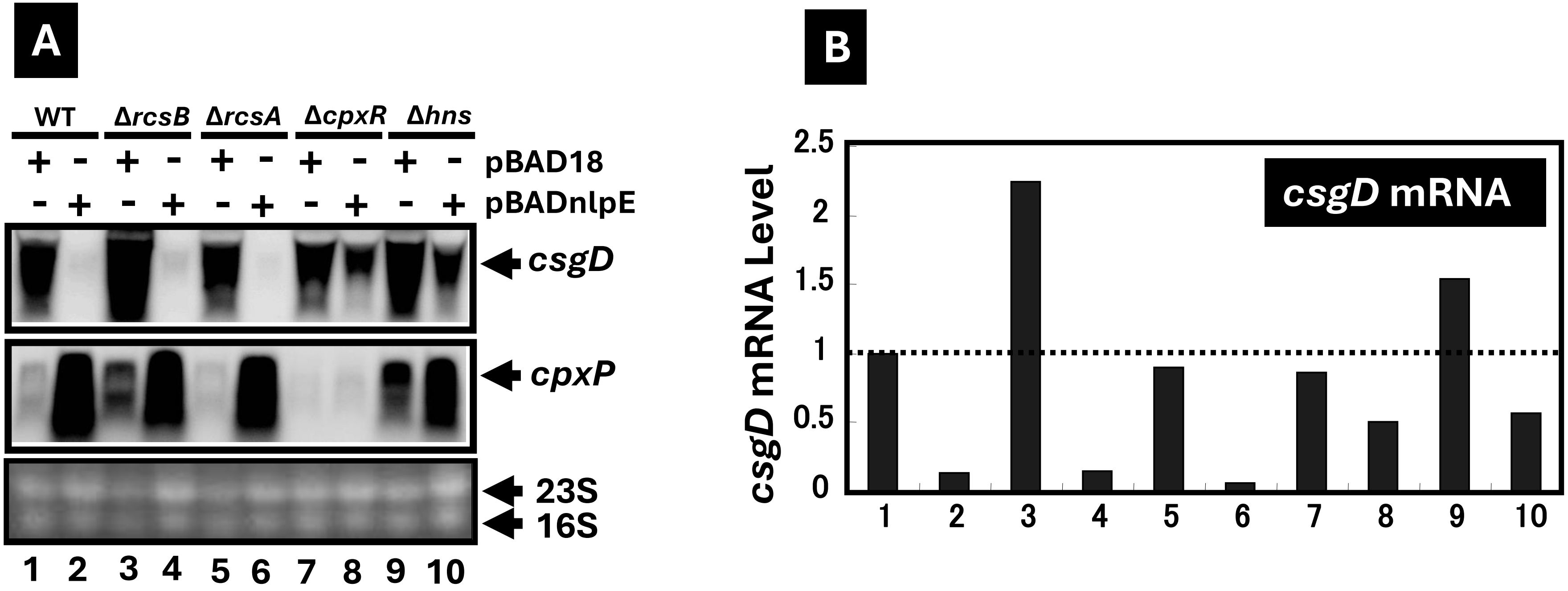

Supplemental Figure S2. Illustrates the impact of CpxP overexpression through Northern blot analysis to assess the expression level of *csgD* mRNA. Panel (A) presents data from various *E. coli* strains: the wild-type strain (BW25113, lanes 1 and 2), the *rcsB* deletion strain (JW2205, lanes 3 and 4), the *rcsA* deletion strain (JW1935, lanes 5 and 6), the *cpxR* deletion strain (JW3883, lanes 7 and 8), and the *hns* deletion strain (JW1225, lanes 9 and 10). Each strain was transformed with either a control plasmid vector (pBAD18, lanes 1, 3, 5, 7, and 9) or an *nlpE* expression plasmid (pBADnlpE, lanes 2, 4, 6, 8, and 10). Cultures were grown in YESCA medium at 28° C for 12 hours with 0.2% arabinose. Total RNA was extracted from each culture, fractionated via agarose gel electrophoresis, and subjected to Northern blotting. Detection of *csgD* and *cpxP* mRNA was performed using DIG-labeled *csgD* (upper panel) or *cpxP* (middle panel) probes, while 23S and 16S rRNA were visualized by ethidium bromide staining (lower panel).
Panel (B) shows the quantification of band intensity, normalized to the *csgD* mRNA level in the wild-type strain containing the control plasmid [lane 1 in (B)].
